# Air Pollution, Autophagy, and Skin Aging: Impact of Particulate Matter (PM_10_) on Human Dermal Fibroblasts

**DOI:** 10.3390/ijms19092727

**Published:** 2018-09-12

**Authors:** Seo-Yeon Park, Eun Jung Byun, Jeong Deuk Lee, Sungjoo Kim, Hei Sung Kim

**Affiliations:** 1 Department of Biomedicine & Health Sciences, The Catholic University of Korea, 222 Banpo-daero, Seocho-gu, Seoul 06591, Korea; gkrud777@gmail.com (S.-Y.P.); sjkyoon@catholic.ac.kr (S.K.); 2 Department of Dermatology, Incheon St. Mary’s Hospital, College of Medicine, The Catholic University of Korea, 222 Banpo-daero, Seocho-gu, Seoul 06591, Korea; peakey89@naver.com (E.J.B.); leejd@olmh.cuk.ac.kr (J.D.L.)

**Keywords:** autophagy, human skin fibroblasts, particulate matter (PM_10_), gene analysis

## Abstract

A World Health Organization (WHO) report from 2016 states that over 3 million people die annually from air pollution, which places air pollution as the world’s largest single environmental health risk factor. Particulate matter (PM) is one of the main components of air pollution, and there is increasing evidence that PM exposure exerts negative effects on the human skin. To see the impact of air pollution on skin aging, we analyzed the effect of PM exposure on human dermal fibroblasts (HDFs) with Western blot, enzyme-linked immunosorbent assay (ELISA), and gene analysis. Cultured HDFs were exposed to PM_10_ at a concentration of 30 µg/cm^2^ for 24 h, and their gene/protein expression of inflammatory cytokines, fibroblast chemical mediators, and autophagy were assessed. A total of 1977 genes were found to be differentially expressed following PM exposure. We observed a significantly increased expression of pro-inflammatory genes interleukin (IL)-1β, IL-6, IL-8 and IL-33 in dermal fibroblasts exposed to PM_10_. Protein expression of IL-6 and IL-8 also significantly increased, which complemented our gene analysis results. In addition, there was a significant increase in cytochrome P450 (CYP1A1, CYP1B1), matrix metalloproteinase (MMP-1, MMP-3) mRNA expression, and significant decrease in transforming growth factor (TGF)-β, collagen type I alpha chain (COL1A1, COL1A2), and elastin (ELN) mRNA expression in PM-exposed dermal fibroblasts. Protein expression of MMP-1 was significantly increased and that of TGF-β and procollagen profoundly decreased, similar to the gene analysis results. Autophagy, an integrated cellular stress response, was also increased while transmission electron microscopy (TEM) analysis provided evidence of PM internalization in the autolysosomes. Taken together, our results demonstrate that PM_10_ contributes to skin inflammation and skin aging via impaired collagen synthesis. Increased autophagy in our study suggests a reparative role of autophagy in HDFs stressed with PM, but its biological significance requires further research.

## 1. Introduction

Aging is the result of a complex interaction of biological, physical, and biochemical processes that cause changes and damage to molecules, cellular function, and organs [1]. Among all organs, aging of the skin is of particular interest because of its psychological and social consequences which can directly impact one’s self-esteem. Skin aging results from a combined action of intrinsic and extrinsic factors [2]. From a preventive point of view, the latter is of greater importance because it can be modified more easily. For decades, extrinsic skin aging was thought to result mainly from solar radiation. There is, however, growing evidence that air pollution significantly contributes to skin aging [2,3,4,5,6,7].

Particulate matter (PM), which includes harmful suspended contaminants in the air, is an important component of air pollution. PM_10_, by definition, is composed of inhalable particles that are less than 10 µm in diameter and generally includes all fractions of PM_10_, PM_2.5_, and ultrafine particles. Ambient PM represents a major environmental threat to building constructions and cultural monuments by corrosion and soiling of the materials [8,9], and also to millions of people worldwide who are exposed. Recent epidemiological studies suggest that PM negatively affect the human skin and exacerbates preexisting skin disease [5,6,7,10,11]. However, there is little information regarding the toxicological mechanisms by which PM affect the skin. In vitro and in vivo studies of the lung and heart have shown a wide-range of biological effects after PM exposure, not only in chronic but also in acute settings. The mechanisms by which the PM exerts its damaging effects are thought to involve oxidative stress and inflammation, both of which are important contributors to skin aging [3,4,12,13]. PM application induces dermal inflammation in barrier intact skin [14]. It is generally accepted that there are two potential pathways in the transdermal delivery of PM: (1) the appendageal route (via hair follicles or sweat ducts), and (2) across the stratum corneum (intracellularly or transcellularly) [15]. PM application itself is also known to cause barrier perturbation [16,17].

Autophagy, a regulated catabolic process, is induced in response to various stress including oxidative stress [18]. During autophagy, the cellular components are sequestered within the vesicles and then delivered to the lysosomes for degradation and recycling of the biogenic components. Thus, autophagy can be viewed as a cellular attempt to survive through stress by the removal of dysfunctional organelles. Skin is one of the first organs to be exposed to PM, but the nature of PM-induced skin damage and its autophagic response is still not fully elucidated. Thus, we investigated the changes in cytokine levels, dermal mediators, and autophagy in cultured human dermal fibroblasts following PM exposure with Western blot, ELISA, and gene analysis.

## 2. Results

### 2.1. Cytotoxic Effects of PM_10_ on Primary Dermal Fibroblasts

**Figure 1:**
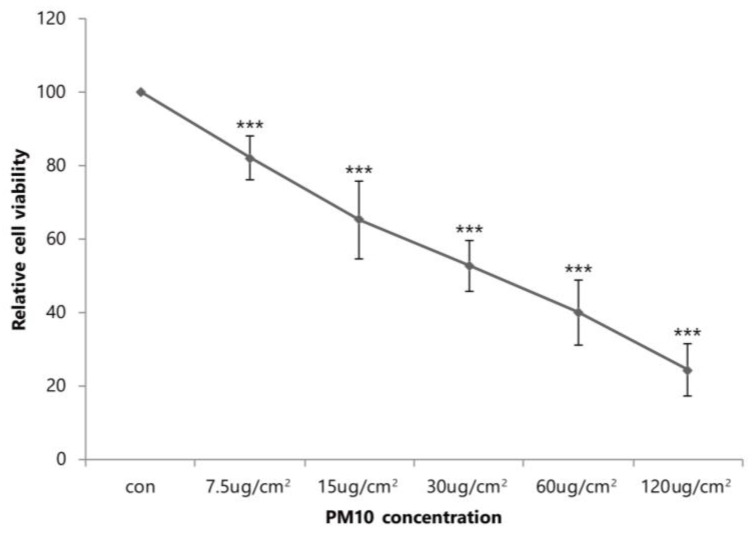
The survival rate of human dermal fibroblasts (HDFs) grown in various concentrations of PM_10_. Data are provided as mean ± SD of three independent experiments. *p*-values were calculated with respect to control by Student’s *t*-test. *p* < 0.001 (***) versus control.

Figure 1 summarizes the survival rate of human dermal fibroblasts (HDFs) grown in various concentrations of PM_10_. Trypan blue exclusion demonstrated a dose-dependent reduction in the number of viable cells. At a concentration of 30 μg/cm^2^, we observed a 47.2% inhibition of cell viability after 24 h compared to control (*p* < 0.001).

### 2.2. The Relationship between PM_10_ and Autophagy in HDFs (Western Blot, Immunochemistry and TEM)

**Figure 2:**
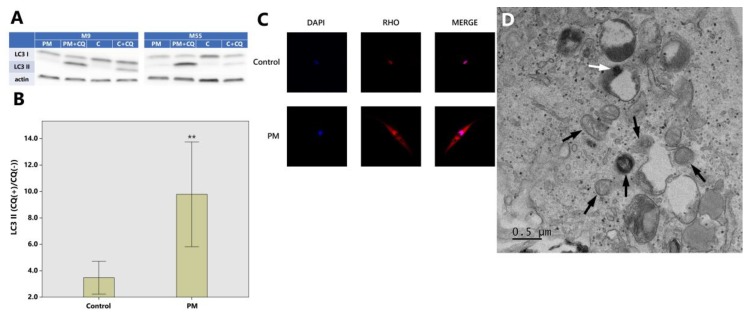
Autophagic degradation is increased in dermal fibroblasts exposed to PM_10_. (**A**) Cell lysates were subjected to SDS-PAGE (sodium dodecyl sulfate-polyacrylamide gel electrophoresis) and Western blotting; PM: particulate matter exposure, PM + CQ: particulate matter + chloroquine exposure, C: control (exposure to nothing), C + CQ: control + chloroquine; LC3: microtubule-associated proteins 1A/1B light chain 3B (a central protein in the autophagy pathway). (**B**) The ratio of LC3-II with and without chloroquine which represent autophagic activity (flux) are 3.47 ± 1.96 (control) and 9.78 ± 6.24 (PM) (Western blot analysis) (** *p* < 0.01). (**C**) LC3 puncta monitored from fluorescence microscopy are increased in PM_10_ treated HDFs compared to control. Merged images were obtained with DAPI (4,6-diamidino-2-phenylindole) (nucleus; blue) and RHO (Alexa Fluor 594, Thermo Fischer Scientific Inc., Waltham, MA, USA) (LC3; red). The images were captured at 20× magnification. (**D**) TEM photo shows deformed mitochondrias (black arrows) and PM internalization in the autolysosome (white arrow) in PM-exposed HDF. As for Western blot analysis, the images are representative of three independent experiments on *n* = 8 sample pairs (PM, control). LC3-II expression is normalized to actin (arbitrary units). Data are provided as mean ± SD. The statistical significance of difference between control and experimental data for autophagy was analyzed using the paired *t*-test. *p*-values < 0.05 were considered statistically significant. Statistical analysis of data was performed using SPSS for Windows software (version 15.0; SPSS Inc., Chicago, IL, USA).

One of the popular methods to measure the autophagic flux is the monitoring of microtubule-associated proteins 1A/1B light chain 3B (LC3) turnover, which is based on the fact that LC3-II is degraded in autolysosomes. If cells are treated with lysosomotropic reagents such as chloroquine which block acidification inside the lysosome or inhibit autophagosome-lysosome fusion, the degradation of LC3-II is blocked, resulting in the accumulation of LC3-II. Accordingly, the differences in the amount of LC3-II between samples in the presence and absence of lysosomal inhibitors represent the amount of LC3 that is delivered to the lysosomes for degradation. Western blot analysis revealed that the ratio of LC3-II with and without chloroquine were 3.47 ± 1.96 (control), 9.78 ± 6.24 (PM) (*p* < 0.01) (Figure 2A,B).

Microtubule-associated proteins 1A/1B light chain 3B (LC3) puncta monitored from fluorescence microscopy were increased in PM_10_ treated HDFs compared to control (no PM exposure) (Figure 2C). TEM revealed structural changes in HDF ultrastructure after PM exposure such as deformed mitochondria and PM internalization in the autolysosomes (Figure 2D).

### 2.3. Effect of PM_10_ on the Expression of Pro-Inflammatory Cytokines and Collagen Metabolism in HDFs (ELISA)

With PM exposure, protein expression of IL-6 and IL-8 increased in HDFs. There was a 1.12-fold upregulation IL-6 (*p* < 0.05), and a 1.17-fold upregulation of IL-8 (*p* < 0.05) in the PM exposed cells compared to controls (Table 1).

PM exposure to HDFs caused an increase in the protein expression of MMP-1 and a decrease in the expression of procollagen and TGF-β. There was a 2.46-fold upregulation of MMP-1 (*p* < 0.05), 1.30-fold downregulation in procollagen (*p* < 0.01), and 1.72-fold downregulation of TGF-β (*p* < 0.01) in the PM exposed fibroblasts compared to controls (Table 1). 

### 2.4. Gene Transcription Profile

**Figure 3:**
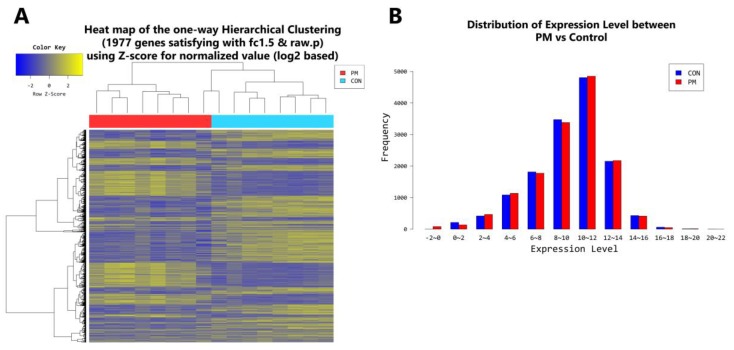

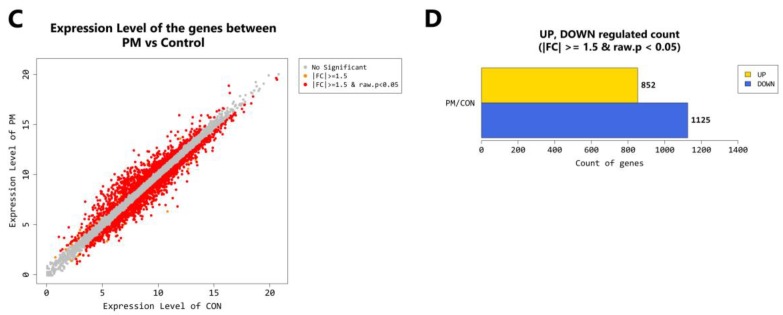
(**A**) Heat map of the one-way hierarchical clustering (PM vs. control). (**B**) Distribution of gene expression level between PM-exposed and control dermal fibroblasts. (**C**) Scatter plot of gene expression level. (**D**) Significant gene count by fold change and *p*-value. *n* = 8 in each group (PM, control). Only those genes exhibiting log_2_ fold change (FC) > 1.5 and *p* < 0.05 were considered differentially expressed genes. For DEG (differentially expressed gene) set, hierarchical clustering analysis was done using complete linkage and Euclidean distance as a measure of similarity.

**Figure 4:**
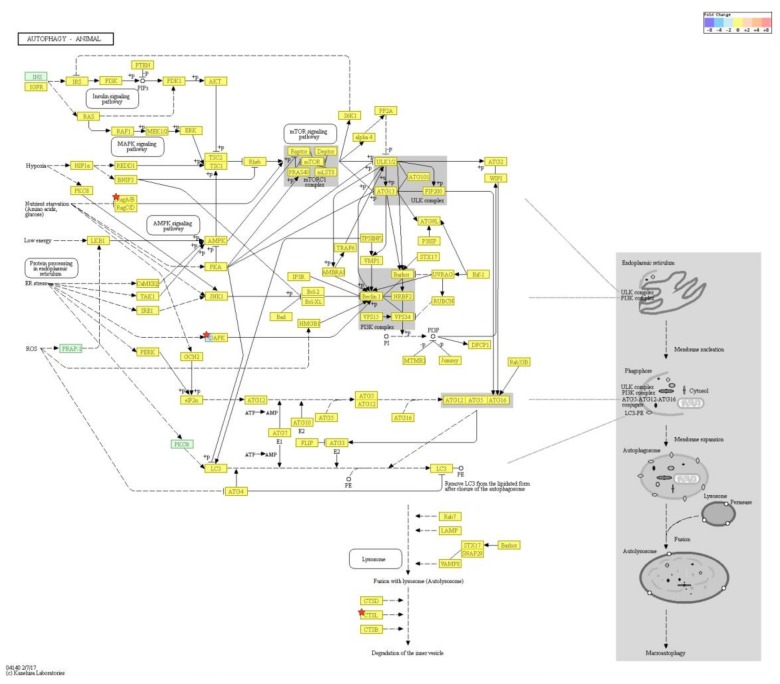
KEGG pathway analysis on autophagy. Among the autophagy-related genes, CTSL was. upregulated, and DAPK1, RRAGB, DAPK2 downregulated in PM exposed cells (*p* < 0.05). Pathway analysis for significant gene list were performed based on KEGG pathway (http://www.genome.jp/kegg/pathway.html) (accessed on 19 July 2018).

**Figure 5:**
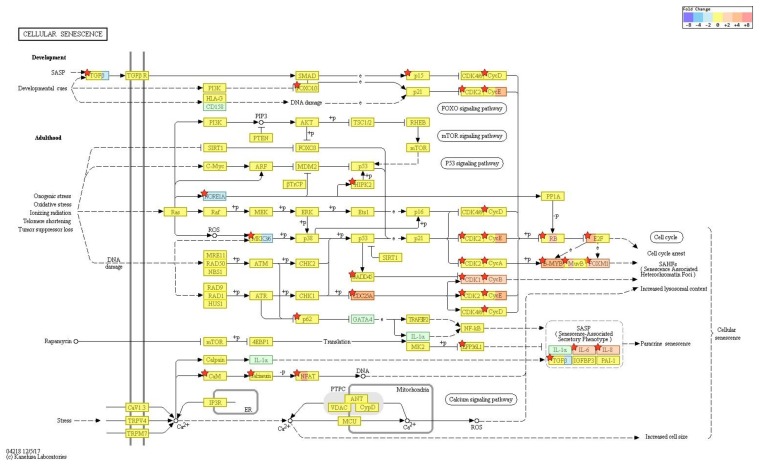
KEGG pathway analysis on cellular senescence. Among the cellular senescence genes, IL-6 and IL-8 expression were increased and TGF-β decreased in PM exposed cells (*p* < 0.05). Pathway analysis for significant gene list were performed based on KEGG pathway (http://www.genome.jp/kegg/pathway.html) (accessed on 19 July 2018).

According to RNA-Seq analysis, a total of 1977 genes were found to be differentially expressed by PM exposure (greater than 1.5-log_2_ folds up and down and a raw *p*-value < 0.05) on whole. Among the 1977 genes, 852 genes were upregulated, and 1125 genes were downregulated (Figure 3A–D). Supervised hierarchical cluster analysis illustrated on the heat map separates PM exposed and control HDFs into two distinct clusters (Figure 3A). From the RNA-sequencing data, we identified an increase in IL-1β, IL-6, IL-8, IL-33, MMP-1, MMP-3, cytochrome P450 (CYP1A1, CYP1B1) and a decrease in TGF-β, collagen type I alpha 1 chain, collagen type I alpha 2 chain, and elastin (Table 2). There was also some change in expression of the autophagy-related genes (Table 3). KEGG pathway analysis on autophagy and cellular senescence is shown in Figure 4 and Figure 5, respectively. Among the autophagy-related genes, CTSL was upregulated (log_2_ fc: 1.575248), and DAPK1 (log_2_ fc: FC: 2.475554), RRAGB (log_2_ fc: 1.524995), DAPK2 (log_2_ fc: 1.818593) downregulated in PM_10_ exposed cells (*p* < 0.05). Among the differentially expressed cellular senescence genes, IL-6 (log_2_ fc: 2.593295) and IL-8 (log_2_ fc: 3.634443) were increased and TGF-β (log_2_ fc: 2.772363) decreased in PM exposed cells (*p* < 0.05).

**Figure 6:**
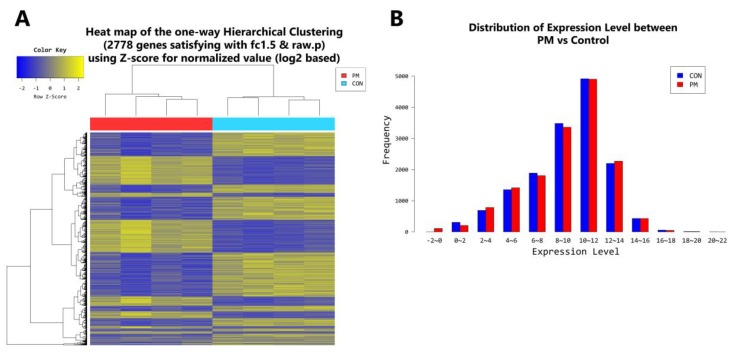

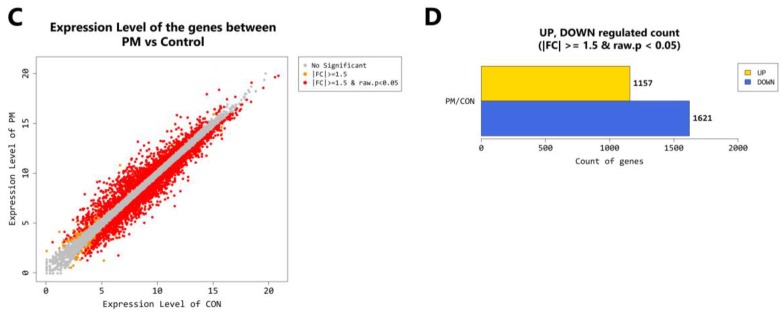
(**A**) Heat map of the one-way hierarchical clustering (PM young vs. control young). (**B**) Distribution of gene expression level between PM-exposed and control young dermal fibroblasts. (**C**) Scatter plot of gene expression level. (**D**) Significant gene count by fold change and *p*-value. *n* = 4 in each group (PM young, control young). Only those genes exhibiting log_2_ fold change (FC) > 1.5 and *p* < 0.05 were considered as differentially expressed genes. For DEG (differentially expressed gene) set, hierarchical clustering analysis was done with complete linkage and Euclidean distance as a measure of similarity.

**Figure 7:**
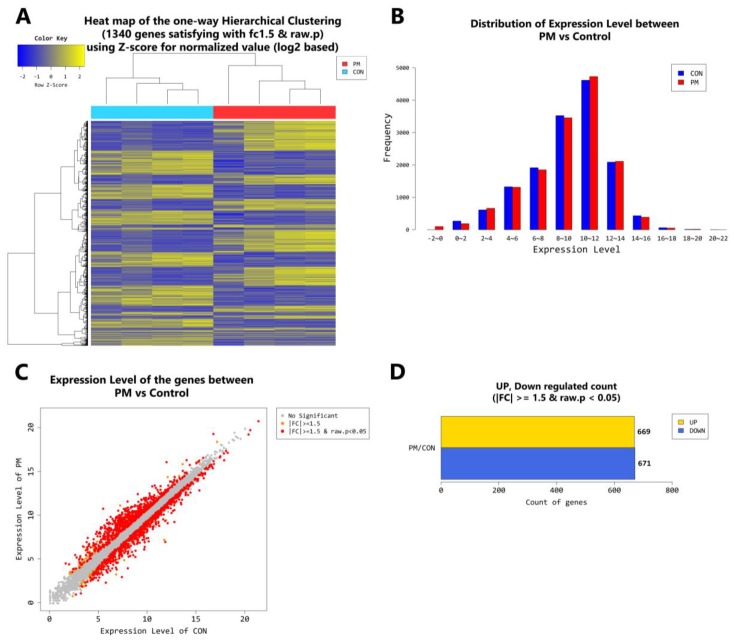
(**A**) Heat map of the one-way hierarchical clustering (PM old vs. control old). (**B**) Distribution of gene expression level between PM-exposed and control old dermal fibroblasts. (**C**) Scatter plot of gene expression level. (**D**) Significant gene count by fold change and *p*-value. *n* = 4 in each group (PM old, control old). Only those genes exhibiting log_2_ fold change (FC) > 1.5 and *p* < 0.05 were considered as differentially expressed genes. For DEG (differentially expressed gene) set, hierarchical clustering analysis was done with complete linkage and Euclidean distance as a measure of similarity.

In the younger population, a total of 2778 genes were found to be differentially expressed by PM exposure (greater than 1.5-log_2_ folds up and down and a raw *p*-value < 0.05). Among the 2778 genes, 1157 genes were upregulated and 1621 genes were downregulated (Figure 6A–D). Supervised hierarchical cluster analysis illustrated on the heat map separates PM exposed and control HDFs into two distinct clusters (Figure 6A). In the older population, a total of 1340 genes were found to be differentially expressed by PM exposure (greater than 1.5-log_2_ folds up and down and a raw *p*-value < 0.05). Among the 1340 genes, 669 genes were upregulated and 671 genes were downregulated (Figure 7A–D). Supervised hierarchical cluster analysis illustrated on the heat map separates PM exposed and control HDFs into two distinct clusters (Figure 7A).

## 3. Discussion

Epidemiologic studies have suggested correlation between increased airborne PM and adverse health effects (e.g., higher risk for cancer and pulmonary and cardiovascular diseases) [19,20]. Recently, there is increasing evidence that ambient PM exposure not only affects the lung and the cardiovascular system but also exerts negative effects on human skin [21]. In this regard, it has been shown that PM exposure increases the risk of inflammatory skin diseases (atopic dermatitis, acne, psoriasis) [12,13,22,23], and influences the development of androgenetic alopecia [24] and skin cancer [25]. As for the impact of PM on skin aging, an increase in soot and particles from traffic was associated with 20% more pigment spots on the forehead and cheeks [5,26]. Yet, the mechanism of how ambient PM causes these effects on the skin are not very well understood.

In this study, we assessed the influence of air pollution on skin aging by exposing human dermal fibroblasts (HDFs) to PM_10_. Skin fibroblasts play a key role in maintaining dermal homeostasis by synthesizing and degrading the extracellular matrix. Dysfunction of dermal fibroblast is closely linked with the formation of deep wrinkles which make them an ideal model for studying extrinsic skin aging.

Observationally, PM is one of the most common components of air pollution. There is evidence that metals in PM cause DNA, protein damage as well as apoptosis of skin cells through the mitochondria-regulated death pathway [27]. Ambient particulates are also capable of inducing skin inflammation. We specifically chose PM_10_ for our study because coarse PM are potentially more hazardous than fine (PM_2.5_) or ultrafine PM (PM_1_), having a substantially higher inflammatory potency [28]. In order to gain insight into the cellular pathways modulated by PM, we performed a series of gene expression analysis on targets known to be involved in skin aging and inflammation.

Oxidative stress is a prominent mechanism by which a variety of toxicants mediate their effects [12,13,29]. Particle-generated oxidants trigger inflammation by activating redox sensitive transcription factors such as NF-κB [30]. Once activated, NF-kB can translocate to the nucleus, and transcribes pro-inflammatory genes. Consistently, we have observed a significantly increased gene expression of NF-κB (FC: 1.677046) as well as IL-1β, IL-6, IL-8 and IL-33 in dermal fibroblasts exposed to PM_10_. Protein expression of IL-6 and IL-8 were also significantly increased, which complemented our gene analysis results.

Activation of the aryl hydrocarbon receptor (AhR) is also proposed to initiate the detrimental effect of PM on the skin [2,4,12,13]. It was recently shown that AhR activation increases MMPs which are responsible for the degradation of collagen and elastin, resulting in skin aging and the formation of wrinkles [4,13]. CYP1A1/CYP1B1, both members of the cytochrome P450 family, are dependent on AhR and are often used as an indicator of AhR activation [4,31,32]. As for our study, there was a significant increase in CYP1A1/CYP1B1, MMP-1, MMP-3 mRNA expression and significant decrease in TGF-β, COL1A1, COL1A2, ELN mRNA expression in PM-exposed dermal fibroblasts which are in line with the findings from prior studies. Protein expression of MMP-1 was significantly increased and that of TGF-β and procollagen profoundly decreased similar to the gene analysis results.

Autophagy serves multiple intracellular recycling roles and acts as a protective cellular response [14]. Interestingly, autophagy has also been proposed as an adaptive mechanism to inflammation [33,34,35]. Cigarette smoke induces both ROS production (oxidative stress) and autophagy, and it was recently demonstrated that cigarette smoke-induced autophagy may have a protective role on cell survival (based on the fact that pharmacological suppression of cigarette-smoke induced autophagy led to a further reduction in cell viability while pharmacological promotion of autophagy increased the viability of the cells under cigarette smoke) [24]. As for our study, a significant number of autophagy-related genes were differentially expressed following PM exposure. Findings from Western blot (LC3II turnover) and immuno-histochemical stain (LC3) show an increase in autophagy which is probably cell-protective against PM-induced inflammation.

In summary, our study provides evidence of potential mechanism that may underlie the cellular effects induced by PM_10_, namely, the modification of mRNA expression. We identified that 1977 genes are differentially expressed in HDFs by PM_10_ exposure. Although our study does not conclusively identify the mechanisms underlying PM_10_ toxicity, oxidative stress generation, activation of AhR and the induction of an inflammatory cascade seem to be the important steps. PM_10_ also triggers autophagy in dermal fibroblasts which seems to be beneficial to their viability. Based on the findings from this study, we will further investigate whether these mRNAs qualify as potential biomarkers of exposure to PM_10_ in humans. The biomarkers can be applied to monitor human exposure to environmental pollutants and clarify their relationship with health outcomes.

From curiosity, we also compared the effect of PM_10_ on young and old fibroblasts. In the young fibroblasts, a total of 2778 genes were found to be differentially expressed by PM exposure (Figure 6), whereas in the older population, the number was significantly lower (1340 genes) (Figure 7). As for the specific genes, PM/control ratio for IL-1β, IL-6, IL-8, IL-33, MMP-3, CYP1A1, COL1A1, and COL1A2 were higher in the young population which imply that PM_10_ exposure may have more profound effects in terms of skin inflammation and aging in the younger population.

## 4. Materials and Methods

### 4.1. Skin Samples

Eight skin samples (all from non-sun-exposed areas, ages 9–94 years old, average 40.5; young skin, *n* = 4, ages 9–11, average: 10.2; old skin, *n* = 4, ages 55–94, average: 70.5) were collected from healthy Korean males within the months of February to May. None of the donors had any medical condition or was under medication.

### 4.2. Ethics

This study was approved by the institutional review board of Incheon St. Mary’s Hospital, The Catholic University of Korea (OIRB-00252-004) (25 January 2016). Eligible patients/guardians were informed about the study protocol in clear, simple language before an informed consent was obtained.

### 4.3. Cell Culture

Human primary skin fibroblasts were isolated from the tissue specimens by enzymatic digestion using collagenase (500 U/mL; GIBCO, Grand Island, NY, USA). Cells were cultured in Dulbecco’s Modified Eagle’s media-high glucose (DMEM; Sigma-Aldrich, St. Louis, MO, USA) supplemented with 10% fetal bovine serum and 1% penicillin/streptomycin at 37 °C in a 5% CO_2_ humidified incubator. All cells used in the present study were obtained from the fourth cell passage. A proportion of cells were treated with chloroquine (50 μM; Sigma) for 4 h before cell preparation.

### 4.4. Particulate Matter Preparation

PM_10_ was collected in 2005 from an air intake filtration system of a major exhibition center in Prague, Czech Republic (National Institute of Standards & Technology, Standard reference Material^®^ 2787). PM suspension was freshly prepared by resuspending PM particles in distilled water at a stock concentration of 30 mg/mL, and vortexing for 30 min at maximum speed.

### 4.5. Cell Viability Test

Cell viability was measured by Trypan blue exclusion assay and was counted using a hemocytometer. The number of viable cells was determined based on their exclusion of 0.4% Trypan blue (Sigma-Aldrich, St. Louis, MO, USA). Briefly, the cells were seeded on 12-well plates at a density of 2 × 10^5^ cells/well, incubated for 24 h in DMEM containing 0 μg/cm^2^ to 120 μg/cm^2^ of PM_10_. The relative cell viability was normalized by the value of control cells incubated without PM and was expressed as mean ± SD of the results from three independent experiments.

### 4.6. Cell Lysis and Western Blotting

Cell pallets were dissolved in RIPA (Radioimmunoprecipitation assay) lysis buffer (1% NP40, 0.5% Na-DOC, 0.1% SDS, 50 mM Tris-HCl with pH 8.8) containing Protease Inhibitor Cocktail (genDEPOT, Houston, TX, USA). Protein concentration was measured using the Bradford assay (Bio-Rad Laboratories, Hercules, CA, USA). The proteins were transferred to a nitrocellulose membrane and put through immunoblotting with antibodies specific for LC3B (Abcam, Cambridge, UK, ab51520) and β-actin (ABM, Vancouver, BC, Canada, G043). A secondary antibody conjugated to horseradish peroxidase (HRP) and the enhanced chemiluminescence (ECL) system (Dong-In Biotech, Seoul, Korea) were used to detect the chemiluminescent signal. Western blot films were scanned on a Cannon Flatbed scanner and then analyzed using an ImageJ gel analysis software (Version 1.44; NIH, Bethesda, MD, USA). Band intensities were quantified and made reference to actin control bands. The Western blot was performed on all 8 sample pairs (control and PM exposed), twice as independent experiments, with cells used at the same passage number.

### 4.7. Enzyme-Linked Immunosorbent Assay (ELISA)

The supernatant media and cells from primary fibroblast culture were collected after treatment with and without 30 μg/cm^2^ of PM_10_ for 24 h. The test for the cytokines IL-6, IL-8 (Qiagen, Hilden, Germany, MEH-004A), pro-collagen (Lsbio, Seattle, WA, USA, LS-F25248), MMP1 (Lsbio, Seattle, WA, USA, LS-F4960), TGF-β1 (Lsbio, Seattle, WA, USA, LS-F1002-1) were performed according to the manufacturer’s instructions. All 8 sample pairs (control, PM exposed) were in triplicate (replicate wells).

### 4.8. Immunochemistry and Confocal Microscopy

After a 24 h incubation with and without 30 μg/cm^2^ of PM_10_, HDFs were washed twice with PBS and then fixed with 4% paraformaldehyde in 1 mL of PBS for 30 min at room temperature. The HDFs were then washed with PBS again, permeabilized with 0.1% Triton X-100 for 20 min, blocked with PBS containing 2% BSA, and then incubated for 1 h with primary antibody against LC3II (Abcam, Cambridge, UK, ac51520). The cells were then washed two times, 10 min per wash, with 2% BSA in PBS and incubated with Alexa Fluor 594 goat anti-rabbit secondary antibody (Thermo Fisher Scientific, Waltham, MA, USA, A11037) for an hour. All images were visualized by confocal microscopy (Zeiss, Gottingen, Germany, LSM 510 Meta) and were transferred to a computer equipped with Zen Light Edition (Zeiss, Gottingen, Germany) for analysis.

### 4.9. Transmission Electron Microscopy (TEM)

The fibroblasts were incubated with and without 30 μg/cm^2^ of PM_10_ for 24 h, then washed 2 times with PBS, and harvested. The cell pallets were fixed with 2.5% glutaraldehyde in 0.1 M PBS at 4 °C overnight and washed with 0.1 M PBS. After immersion in 2% agarose gel, the cells were post-fixed in 4% osmium tetroxide solution for an hour. After washing with distilled water, the cells were dehydrated in a graded series of ethanol (30%, 60%, 70%, 90%, and 100%), stained with 0.5% uranyl acetate (1 h), and embedded in epoxy resin. The resin was polymerized at 60 °C for 2 days. Ultra-thin sections prepared with an ultra-microtome were then stained with 5% aqueous uranyl acetate and 2% aqueous lead citrate. After air dry, the slides were observed under the TEM (JEOL, Tokyo, Japan, JEM-2100).

### 4.10. mRNA-Seq

In constructing cDNA libraries with the TruSeq RNA library kit, a total of 1 µg of RNA was used. The protocol consisted of polyA-selected RNA extraction, RNA fragmentation, random hexamer primed reverse transcription and 100 nt paired-end sequencing by Illumina HiSeq4000 (San Diego, CA, USA). The libraries were quantified with qPCR based on the qPCR Quantification Protocol Guide and qualified using an Agilent Technologies 2100 Bioanalyzer (Santa Clara, CA, USA).

We preprocessed the raw reads and aligned the processed reads to the *Homo sapiens* (*hg19*) using HISAT v2.0.5 (https://ccb.jhu.edu/software/hisat2/) [36]. HISAT utilizes two types of indexes for alignment (a global, whole-genome index and tens of thousands of small local indexes) and generates spliced alignments that is several times faster than Bowtie and BWA. The reference genome sequence of *Homo sapiens* (*hg19*) and annotation data were downloaded from the UCSC table browser (http://genome.uscs.edu) (12 May 2018). After alignment, StringTie v1.3.3b (http://ccb.jhu.edu/software/stringtie/) (12 May 2018) was used to assemble aligned reads into transcripts and to estimate their abundance as FPKM values (Fragments Per Kilobase of exon per Million fragments mapped) [37,38]. FPKM values are normalized with respect to library size and are used for comparison analysis of differentially expressed genes between samples. A total of 8 sample pairs (control and PM exposed; 4 young and 4 old) were examined.

## Figures and Tables

**Table 1:** Effect of PM_10_ on the protein expression of pro-inflammatory cytokines and collagen metabolism in HDFs. ELISA results obtained from PM exposed fibroblasts and their control fibroblasts are expressed in relative ratios (fold change). The significant of the values were identified using the one-sample *t*-test. *p*-values < 0.05 were considered statistically significant. Statistical analysis of data was performed using SPSS for Windows software (version 15.0; SPSS Inc., Chicago, IL, USA).

	PM_10_ Exposed HDFs/PM Non-Exposed (Control) HDFs	*p*-Value
IL-6 ratio	1.12 ± 0.05	*p* = 0.046
IL-8 ratio	1.17 ± 0.05	*p* = 0.037
MMP-1 ratio	2.46 ± 0.89	*p* = 0.027
Procollagen ratio	0.77 ± 0.12	*p* = 0.001
TGF-β ratio	0.58 ± 0.25	*p* = 0.002

**Table 2:** Major genes which were overall differently expressed (greater than 1.5-log_2_ folds up and down and a raw *p*-value < 0.05) in PM-exposed HDFs compared to their control fibroblasts (*n* = 8 sample pairs, all from non-sun exposed areas, ages 9–94 years old, average 40.5) (PM/control. Log_2_ fc) are shown. The impact of PM on the gene expression of young (*n* = 4 sample pairs, ages 9–11, average 10.2) (PM/control. Log_2_ fc (young)) and old (*n* = 4 sample pairs, ages 55–94, average: 70.5) HDFs (PM/control. Log_2_ fc (old)) are also separately presented. Differentially expressed genes were analyzed using the estimates of abundances for each gene in samples. Genes were excluded if they had at least one zero FPKM values in the samples. To facilitate log_2_ transformation, 1 was added to each FPKM value of filtered genes. Filtered data were log_2_-transformed and submitted to quantile normalization. Statistical significance of the differential expression data was determined using independent *t*-test and fold change in which the null hypothesis was that there is no difference among groups. False discovery rate (FDR) was controlled by adjusting the *p*-value with the Benjamini–Hochberg algorithm. Log_2_ fc (fold change): log ratio with the logarithm to base 2 applied.

	PM/Control. Log_2_ fc	PM/Control. Log_2_ fc (Young)	PM/Control. Log_2_ fc (Old)
IL-1β	4.678166	8.732704	1.772352
IL-6	2.593295	4.143132	1.463454
IL-8 (CXCL8)	3.634443	4.527864	2.403414
IL-33	3.237957	3.337679	2.863740
MMP-1	3.189445	2.380204	3.166181
MMP-3	2.106478	6.295658	1.533143
CYP1A1 (Cytochrome P450 1A1)	4.844863	5.736596	2.106431
CYP1B1 (Cytochrome P450 1B1)	3.452584	2.917612	3.750746
TGF-β	−2.772363	−1.168725	−2.549271
COL1A1 (Collagen type I alpha 1 chain)	−2.022854	−1.938849	−1.915619
COL1A2 (Collagen type I alpha 2 chain)	−1.800164	−1.818843	−1.666592
ELN (Elastin)	−2.644748	−2.094849	−3.379390

**Table 3:** Gene array analysis; autophagy-related genes altered (greater than 1.5-log_2_ folds up and down and a raw *p*-value < 0.05) by PM exposure (*n* = 8 sample pairs, all from non-sun exposed areas, ages 9–94 years old, average 40.5). Differentially expressed genes were analyzed using the estimates of abundances for each gene in samples. Genes were excluded if they had at least one zero FPKM values in the samples. To enable log_2_ transformation, 1 was added to each FPKM value of filtered genes. Filtered data were log_2_-transformed and submitted to quantile normalization. Statistical significance of the differential expression data was identified using independent *t*-test and fold change in which the null hypothesis was that no difference exists among groups. False discovery rate (FDR) was controlled by adjusting the *p*-value with the Benjamini–Hochberg algorithm.

RRAGB	SNRPB	DAPK2	CHAF1B	KIAA1549L	CAPS
ADAMTS7	SNRPD1	ABL2	OBSCN	LMCD1	SRPX
LRRK2	U2AF1	HK2	MAP1LC3A	ITGB4	SQSTM1
DAPK1	ZC3H12A	REP15	RAB33A	MAP1A	FAM131B
FOXO1	ALPK1	TMEM150C	ATP1B1	PRKN	PFKP
MTCL1	DPF3	PLOD2	RFWD3	KIAA1324	CHAF1B
DAPK2					

## Abbreviations

PM	Particulate matter
HDF	Human dermal fibroblast
IL	Interleukin
CYP1A1	Cytochrome P450 family 1 subfamily A member 1
CYP1B1	Cytochrome P450 family 1 subfamily B member 1
MMP	Matrix metalloproteinase
TGF	Transforming growth factor
COL1A1	Collagen type 1 alpha 1 chain
COL1A2	Collagen type 1 alpha 2 chain
ELN	Elastin
TEM	Transmission electron microscopy
LC3	Microtubule-associated protein 1 light chain 3
CTSL	Cathepsin L
DAPK1	Death associated protein kinase 1
RRAGB	Ras related GTP binding B
DAPK2	Death associated protein kinase 2
AhR	Aryl hydrocarbon receptor
ROS	Reactive oxygen species
SNRPB	Small nuclear ribonucleoprotein polypeptides B and B1
CHAF1B	Chromatin assembly factor 1 subunit B
KIAA1549L	KIAA 1549 like
CAPS	Calcyphosine
ADAMTS7	ADAM metallopeptidase with thrombospondin type 1 motif 7
SNRPD1	Small nuclear ribonucleoprotein D1 polypeptide
ABL2	ABL proto-oncogene 2, non-receptor tyrosine kinase
OBSCN	Obscurin, cytoskeletal calmodulin and titin-interacting RhoGEF
LMCD1	LIM and cysteine rich domains 1
SRPX	Sushi repeat containing protein, X-linked
LRRK2	Leucine rich repeat kinase 2
U2AF1	U2 small nuclear RNA auxiliary factor 1
HK2	Hexokinase 2
MAP1LC3A	Microtubule associated protein 1light chain 3 alpha
ITGB4	Integrin subunit beta 4
SQSTM1	Sequestosome 1
ZC3H12A	Zinc finger CCCH-type containing 12A
REP15	RAB15 effector protein
RAB33A	RAB33A, member RAS oncogene family
MAP1A	Microtubule associated protein 1A
FAM131B	Family with sequence similarity 131 member B
FOXO1	Forkhead box O1
ALPK1	Alpha kinase 1
TMEM150C	Transmembrane protein 150C
ATP1B1	ATPase NA+/K+ transporting subunit beta 1
PRKN	Parkin RBR E3 ubiquitin protein ligase
PFKP	Phosphofructokinase, platelet
MTCL1	Microtubule crosslinking factor 1
DPF3	Double PHD fingers 3
PLOD2	Procollagen-lysine, 2-oxoglutarate 5-dioxygenase 2
RFWD3	Ring finger and WD repeat domain 3
KIAA1324	KIAA1324
